# Optimizing outcomes in ear surgery: A dynamic framework for assessing postoperative complications and quality of care

**DOI:** 10.1007/s00405-025-09740-y

**Published:** 2025-10-15

**Authors:** Susanne Isabella Günther, Joshua Böhrenz, Theresa Lüdke, Marie- Luise Polk, Max Kemper, Thomas Zahnert, Marcus Neudert

**Affiliations:** Department of Otorhinolaryngology, Head and Neck Surgery, Ear Research Center Dresden (ERCD), Faculty of Medicine and University Hospital Carl Gustav Carus, TUD Dresden University of Technology, Fetscherstraße 74, 01307 Dresden, Germany

**Keywords:** Ear Surgery, Postoperative Complications, Quality Indicators, Health Care, Registries

## Abstract

**Purpose:**

To ensure continuous, standardized documentation of postoperative courses after ear surgery. This partial analysis focuses on quantifying the occurrence and frequency of specific complications. A complication registry was implemented to provide quality indicators, facilitating early detection and dynamic assessment.

**Methods:**

In this prospective, single-centre cohort study, 2,120 patients who underwent primary or revision middle ear surgery (Jan 2019 – Jun 2022) were entered in the registry and evaluated. Complications were assessed during regular follow-up visits and analysed dynamically using the Kaplan–Meier estimator, introducing the Complication Persistence Function (CPF) as a novel metric.

**Results:**

A total of 528 (24.9%) complication-related cases involving 570 distinct complications were documented. The introduction of the CPF allowed for the first time the characterisation of the regression time of complications by extracting parameters such as the median persistence time (MPT), the plateau onset time (POT) and the persistence rate (PR). Bone conduction (BC) threshold shift and facial nerve palsy (FNP) were analysed in detail. Immediate and late-onset subgroups of BC threshold shift and FNP showed significantly different recovery dynamics (p < 0.05).

**Conclusion:**

This study introduces a standardised, dynamic approach to the documentation and analysis of otosurgical complications. The CPF provides robust metrics for assessing surgical outcome quality and should be considered a complementary method alongside traditional outcome measures. These findings can help establish mandatory documentation parameters and improve comparability across clinical centres.

## Introduction

Despite strict adherence to evidence-based surgical guidelines, unforeseen outcomes and complications remain an inherent risk of middle-ear surgery. Reported postoperative events span a spectrum from minor, self-limiting issues—such as transient vertigo or reversible bone-conduction (BC) threshold shifts—to major sequelae, including facial nerve palsy, permanent hearing loss, and meningitis [1–4].

A universally accepted framework for reporting such complications is lacking [4]. Current studies vary in both the timing of postoperative assessments and the length of follow-up, although complication profiles are time-dependent: early incidences decline as transient events resolve, while a smaller proportion persists or emerges late [5]. Furthermore, there is no universally accepted classification system distinguishing between mild and severe complications [4]. Internationally recognized definition criteria for functional impairments such as the deterioration of BC threshold would be particularly beneficial. Establishing a standardised minimum reporting framework for complications after middle ear surgery, analogous to the existing minimum standards for surgical outcomes, would facilitate consistency in reporting and data interpretation [6, 7]. The absence of such standards complicates the evaluation of existing literature, making meaningful comparisons between studies difficult or nearly impossible.

The majority of studies on this topic rely on retrospective data analyses, which preclude the subsequent inclusion of complications that were not initially documented [8–11]. Continuous, prospective data collection is essential to capture late-onset complications and to accurately describe the dynamic course of complications, both in terms of frequency and severity. Furthermore, a significant proportion of studies address a restricted range of complications, impeding a thorough evaluation of rare yet potentially substantial adverse events [8, 12].

It is imperative to establish a clear distinction between surgical complications and instances of malpractice. Only by making this distinction can an open discourse on complications occur without fear of legal repercussions. By definition, a complication is an adverse effect or exacerbation of a disease state caused by unforeseen circumstances or events [13–15]. A complication is thus a deviation from the expected postoperative course as recognized by the treating physician. However, current literature lacks standardised definitions for many relevant parameters. The terms *mistake* (failure of a planned action or implementation of an incorrect plan) and *failure to cure* (persistence of a disease or condition despite surgical intervention) [14] should be clearly delineated. Importantly, the occurrence of a complication assumes that the surgical procedure was conducted with technical, professional, ethical, and moral integrity.

Finally, the absence of complications and failures to cure can serve as an indirect measure of treatment quality [4]. Following this paradigm shift, systematically recorded and documented complications should be recognized as complementary outcome parameters in surgical evaluation. The present study aims to contribute to this discourse by summarizing both established and novel quality indicators that can directly or indirectly assess the quality of surgical care in middle ear surgery.

## Methods

The present analysis represents a partial evaluation of a prospective cohort study on the systematic documentation of postoperative complications after ear surgery. The primary objective of both the overall study and this partial analysis was the incidence and frequency of specific complications following ear correction. The secondary outcomes included the dynamic course and persistence of the individual complications. Epidemiological parameters such as comorbidities or the type of ear surgery as well as data on the severity of the complications are explicitly not included in this report, as the focus is on methodological recording. The study was approved by the local ethics committee (BO-EK-157042022; institution name blinded for review) and was conducted in accordance with the Declaration of Helsinki.

### Ear surgery registry

In the course of this longitudinal observational study, all patients who have undergone ear surgery at a tertiary referral center since 1 st January 2019 were systematically evaluated. From this date onward, all patients were prospectively registered in a database along with their respective surgery dates. Pre-existing institutional data were retrospectively collected to identify and define relevant complications for systematic prospective documentation in routine clinical practice.

The study included patients undergoing middle ear surgery for chronic otitis media (with or without cholesteatoma), hearing-improving procedures (tympanoplasty, stapesplasty, active middle ear implants, cochlear implants), and vestibular schwannoma surgery. Revision surgeries were excluded from the dataset if the initial surgery had been performed at our institution, as these cases were already registered in the database. In patients undergoing bilateral surgery, each ear was documented as an independent case.

During each postoperative follow-up visit, patients were routinely assessed for the presence of complications. Any identified complications were systematically recorded in the database using a dichotomous (yes/no) classification system.

### Regular follow-up timing

Routine assessments comprised a clinical examination and audiometric measurements, which were conducted in the following sequence: preoperatively, on postoperative day 1, between days 14 and 56 (2 to 8 weeks), between days 57 and 84 (8 to 12 weeks), and additionally if deemed necessary by the treating physician. On the first postoperative day, only the retroauricular bone-conduction threshold was measured; at all subsequent time points, however, both the bone and air conduction thresholds were measured using a clinical audiometer (Auritec AT900). Postoperative follow-up continued for a minimum period of six months and a maximum period of 24 months to ensure complete wound healing.

Beyond complications, patient-related data—including laboratory parameters and pre-existing medical conditions—were retrospectively extracted from the electronic hospital information system and systematically recorded in an anonymised registry. The retrospective collection of these baseline data facilitated cross-validation of the complication documentation against clinical records, ensuring completeness. As all patients undergoing ear surgery were prospectively enrolled, data integrity and comprehensiveness were guaranteed.

### Definition, classification and quantification of ear-specific complications

Complications were defined as any unplanned and/or unexpected negative deviation from the preoperative (baseline) condition that impairs, complicates, or prevents the normal healing process [13]. The characteristics of ear-specific complications have been described previously [4] and are briefly summarized in Table 1.

**Table 1:** Ear specific major and minor complications after ear surgery

Major complications	
	BC threshold shift ≥ 15 dB in ≥ 3 frequencies or ≥ 20 dB in ≥ 2 frequencies
	New onset facial nerve palsy
	Vertigo with stimulus/failure nystagmus
	New onset Tinnitus with BC threshold shift
	Wound healing disorders with necessary surgical revision
	(Rhino-) liquorrhea
	Intracranial complications (e.g. postoperative meningitis)
Minor complications	
	Vertigo without stimulus/failure nystagmus
	New onset Tinnitus without BC threshold shift
	Wound healing disorders without necessary surgical revision

Based on this definition, systematic recording was initially performed dichotomously as either present or absent. All complications, with the exception of bone-conduction threshold shifts, were documented on the basis of clinical examination and symptom assessment. BC threshold shift was defined as a decrease of ≥ 15 dB at ≥ 3 frequencies or ≥ 20 dB at ≥ 2 frequencies on pure-tone audiometry. Facial nerve palsy (FNP) was classified according to the House-Brackmann scale (HBS) [16], as no validated classification systems exist for other complications.

For this study, BC threshold shift and FNP were analysed in detail, although all complications were systematically recorded in the registry.

Complications were categorized into "immediate" (occurring within 48 h postoperatively) and "late" (occurring beyond 48 h) [4]. Immediate complications are typically direct consequences of the surgical intervention (e.g., intraoperative trauma), whereas late complications result from secondary processes such as wound healing disturbances, infections, or postoperative swelling, often unrelated to direct surgical manipulation.

### Characterisation and dynamics of complications

Once a complication was identified during routine follow-up or an unscheduled complication-related visit, the corresponding case was flagged in the registry. From that point onward, all complication-specific parameters were continuously monitored until either a normal or a predictably persistent postoperative status was achieved. Consequently, complications were no longer assessed as static dichotomous variables but rather as dynamic entities that evolved over time. Statistical analyses were employed to define and quantify these parameters.

### Complications after ear surgery as unmet medical need (UMN)

The definition of a UMN is contingent upon the absence of a satisfactory method of prevention, diagnosis, or treatment, and the presence of a significant individual and/or societal impact [17]. The identification of a UMN is achieved through the formulation of key questions, which are derived from the UMN dimensions [18]. The aforementioned three dimensions encompass inquiries pertaining to treatment alternatives, the severity of the disease, and the patient population. The absence of standardised characterisation and dynamic description of ear-specific complications following surgical procedures must be comprehended within the context of these fundamental inquiries as an unmet medical need (UMN).

### Complications Persistence Function (CPF) and specific values

**Fig. 1:**
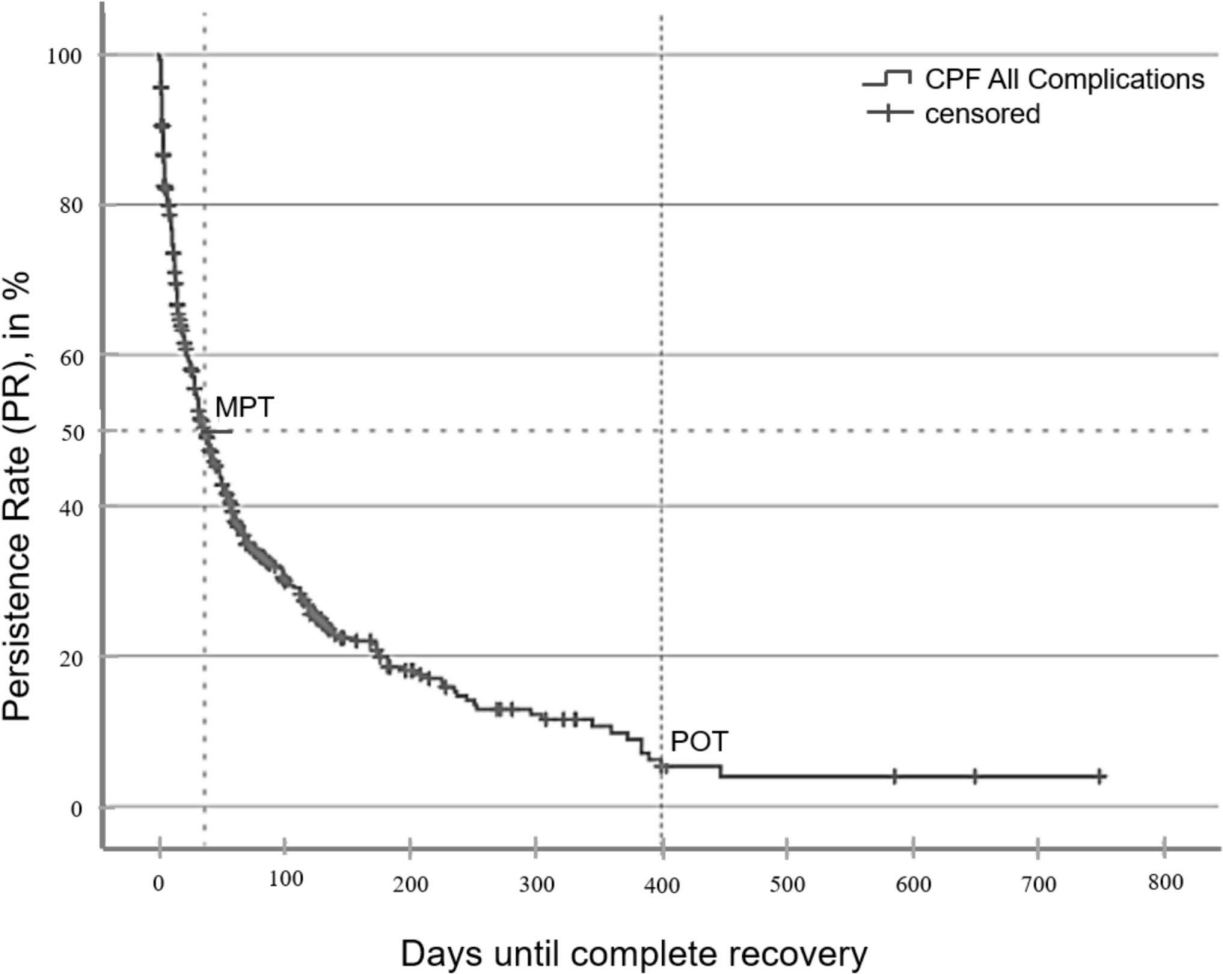
Complications Persistence Function (CPF) showing recovery from complications: The curve represents the proportion of patients with persisting complications over time. At baseline (day 0), 100% reflects the total number of complications observed (n = 570). Once full recovery (restitutio ad integrum) occurs, the corresponding case is removed from the denominator, and the persistence rate decreases accordingly. Censored cases indicate patients who had not fully recovered by the end of follow-up. MPT (Median Persistence Time) marks the time point at which 50% of complications had resolved; POT (Plateau Onset Time) indicates the point beyond which no further recoveries were observed (= Persistence Rate (PR))

The Kaplan–Meier estimator was employed to describe the absolute frequency of complications over time. Once a complication resolved and *restitutio ad integrum* was achieved, there was a corresponding decrease in the frequency count. This allowed for the visualization of complication persistence as a declining curve, referred to as the Complication Persistence Function (CPF) (Fig. 1).

From the CPF, key complication-specific values were extracted:

- Median Persistence Time (MPT): The time point (in days) at which 50% of complications have resolved.
- Plateau Phase Onset Time (POT): The time point at which no further changes in complication frequency are observed.
- Persistence Rate (PR): The proportion of complications that remain at the onset of the plateau phase.

The observed persistence rate (OPR) differs from the PR in that it represents the actual number of patients with persistent complications at the final follow-up.

### Analysis of two selected complications

Two complications—BC threshold shift and FNP—were analysed in detail alongside the overall assessment of all recorded complications.

BC threshold shift was defined as a reduction in BC threshold of ≥ 15 dB at ≥ 3 frequencies or ≥ 20 dB at ≥ 2 frequencies (postoperative vs. preoperative). Once identified, the deterioration was quantified as the pure-tone average (PTA) difference (BC:PTAΔ), calculated as:

$$BC: PTA\Delta =BC:{PTA}_{\mathrm{post}}-\mathrm{BC}:{\mathrm{PTA}}_{\mathrm{pre}}$$

PTA values were computed for two frequency ranges:

- BC: PTA_4_: 0.5, 1, 2, and 4 kHz
- BC: PTA_all_: 0.25, 0.5, 1, 2, 3, 4, and 6 kHz

Facial Nerve Palsy (FNP): FNP was graded using the House-Brackmann Scale (HBS), where grade 1 indicates normal function and grade 6 denotes complete paralysis [16].

### Statistical methods

All statistical analyses were conducted using Microsoft Excel 2010 (Microsoft Corporation, Redmond, WA, USA) and IBM SPSS Statistics 27 (SPSS Inc., Chicago, IL, USA). Pre- and post-operative mean differences were analysed using one-way (or multi-way) repeated measures analysis of variance (ANOVA), with post-hoc pairwise comparisons. Unless otherwise specified, results are expressed as mean ± standard deviation (SD). The Kaplan–Meier estimator was used to determine the probability of full symptom resolution over time. Post hoc comparisons were conducted using the log-rank test, and subgroup assignments were performed in a post hoc manner. A p-value of < 0.05 (*) was considered statistically significant.

## Results

Since the inception of the registry, n = 2,376 patients (54% male, 46% female) were enrolled up until 31 st December 2022. The mean patient age was 45 ± 23 years (range: 0–96 years). As the registry includes all patients undergoing ear or ear-related lateral skull base surgery—not just those with complications—this number represents the entire surgical cohort. The baseline characteristics of the patient population remained stable over the years.

### Incidence and frequency distribution of all complications.

A total of 528 (24.9%) complication-related cases were documented, comprising 570 different complications following 2,120 ear surgeries performed between 1 st January 2019 and 30th June 30 2022. This indicates that one or more complications—either major or minor—occurred at some point postoperatively, either immediately or at a later stage.

### Quantification of ear-specific complications

Table 2 provides a summary of the incidence, frequency, and progression of individual complications based on prospective data collection over a 24-month follow-up period. When considering the dynamic course of complications, the raw frequency of occurrence must be interpreted in a more differentiated manner.

**Table 2:** Occurrence, frequency and course of individual complications

		Complications after ear surgery (n = 2120)		fully regressed		partially regresssend or persistend (OPR^*a*^)	
		n	%	n	%	n	%
Major complication		467	22.0	321	15.1	159	7.5
BC threshold shift	total	290	13.6	194	9.2	96	4.5
	immediate	182	8.6	127	6.0	55	2.6
	late	108	5.1	67	3.2	41	1.9
Facial nerve palsy	total	47	2.2	33	1.6	16	0.8
	immediate	29	1.4	16	0.8	13	0.6
	late	18	0.8	15	0.7	3	0.1
Vertigo with nystagmus	total	102	4.8	68	3.2	34	1.6
	immediate	92	4.3	61	2.8	31	1.5
	late	10	0.5	7	0.3	3	0.1
Wound healing disorders with necessary surgical revision	total	15	0.7	15	0.7	-	-
(Rhino-) liquorrhoea	total	13	0.6	13	0.6	-	-
		n	%	n	%	n	%
Minor complication		75	3.5	79	3.7	3	0.1
Vertigo without nystagmus	total	69	3.3	66	3.1	3	0.1
	immediate	50	2.3	49	2.3	1	0.05
	late	19	0.9	17	0.8	2	0.1
Wound healing disorders without necessary surgical revision	total	41	1.9	41	1.9	-	-
Total		570	26.7	415	19.6	161	7.6

^*a*^* OPR, observed persistence rate*

For instance, 296/2,120 (13.9%) patients developed a BC threshold shift postoperatively. However, by the time of analysis, 194/2,120 (9.2%) cases had shown complete or partial regression, while 102/2,120 (4.8%) cases remained unchanged.

Facial nerve involvement was also observed. Of the 49 cases (2.3%) identified, 33 cases showed complete or partial recovery during the observation period. With continued follow-up, further regression of the remaining partial paresis is anticipated.

### Characterization of a complication course

A rapid decrease in the number of complications was observed during follow-up visits. As illustrated in Fig. 1, 50% of the initial complications had been resolved within 36 ± 3.6 days (MPT: 36 ± 3.6 days, 95% CI [28.9; 43.1]). The plateau phase was reached at 400 days, beyond which no further changes in complication frequency were observed (POT: 400 days). At this time, only 157 complications (27.5% of complications, 8.1% of all cases) remained unresolved, corresponding to an observed persistence rate (OPR) of 7.4%.

### Difference between calculated and observed persistence

Patients with unresolved complications at the final observation time point are censored in the Complication Persistence Function (CPF), regardless of the exact postoperative interval. Although the actual persistence status of these complications remains unknown, the CPF enables statistical estimation of their likelihood of persistence. Based on this model, 140 patients (5%) are expected to have a persistent complication at 400 days. This 5% represents the calculated persistence rate (PR), which differs from the observed PR (OPR) by 17 patients.

### Plateau Onset Time (POT) and minimum follow-up

The plateau onset time (POT) determined by the Complication Persistence Function (CPF)—400 days in the present cohort—serves as a statistical benchmark for the minimum required follow-up. This threshold is essential for distinguishing transient events from truly persistent complications within the study period.

To evaluate persistence accurately, the patient-inclusion window should end at least 400 days before the final data-collection point. A key advantage of the CPF is its ability to statistically simulate recovery from complications that occur at different postoperative intervals and to estimate long-term outcomes, even when individual follow-up times vary.

**Fig. 2:**
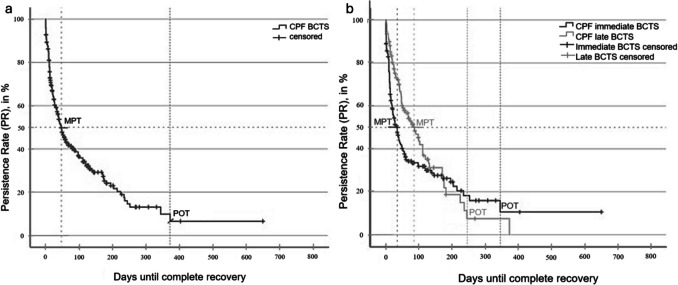
**a** Complications Persistence Function (CPF) of bone-conduction threshold shift (BCTS) (n = 296) **b** CPF comparing immediate (n = 182) and late (n = 108) BCTS (p < 0.05). POT: plateau onset time

**Fig. 3:**
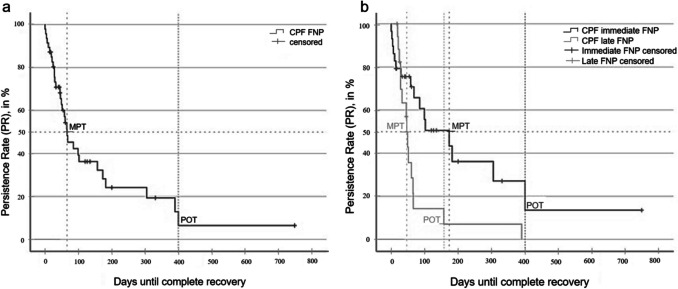
**a** CPF of facial nerve palsy (FNP) (n = 47) **b** CPF comparing immediate (n = 29) and late (n = 18) FNP (p < 0.05). *CPF: complication persistent function; MPT: median persistence time; POT: plateau onset time; PR: persistence rate*

Because complication profiles differ, a differentiated analysis was performed. Table 3 summarises the key parameters for BC threshold shift and facial-nerve palsy (FNP), and the corresponding CPF curves are presented in Figs. 1, 2 and 3.

**Table 3:** Characteristic and complication-specific values

		Frequency, n (%)	MPT^*b*^, days ± SD	POT^*c*^, days	PR^*d*^, %	OPR^*a*^, %
Included surgeries		2120 (100)				
All complications		528 (24.9)	36 ± 3.6	400	4.0	27.5
BC^e^ threshold shift		296 (14.0)	48 ± 5.5	371	6.0	33.3
	immediate	182 (8.6)	35 ± 5.6	345	10.0	30.2
	late	108 (5.1)	86 ± 17.2	243	8.0	38.0
FNP^f^		48 (2.3)	66 ± 17.1	400	10.0	35.4
	immediate	29 (1.4)	173 ± 68	400	14.0	44.8
	late	18 (0.8)	46 ± 3.6	157	7.0	16.7

^*b*^*MPT, median persistence time; *^*c*^*POT, plateau phase onset time; *^*d*^*PR, persistence rate; *^*a*^*OPR, observed persistence rate; *^*e*^*BC, bone-conduction; *^*f*^*FNP, facial nerve palsy*

### Analysis of Bone-Conduction (BC) threshold shift

The median persistence time (MPT) for BC threshold shift was 48 ± 5.5 days (95% CI, 37–59 days; Fig. 2a). At the last clinical contact, 33.3% of cases had not yet fully recovered, which means that the observed persistence rate (OPR) is well above the modelled persistence rate (PR) of 6% (approx. 18 patients).

Among the 185 episodes of immediate-onset BC threshold shift, 55 (2.6%) were still unresolved at the final follow-up, whereas 41 (2.0%) of 108 late-onset episodes remained persistent. The median persistence time in the immediate-onset cohort was 51 days shorter than in the late-onset cohort (p < 0.05; Fig. 2b), underscoring the distinct recovery trajectories of early- versus late-onset BC threshold shift.

### Analysis of Facial Nerve Palsy (FNP)

The median persistence time (MPT) for FNP was 66 ± 17.1 days (95% CI, 32—99 days; Fig. 3a). At the last follow-up, 17 patients (0.8%) had not yet achieved full recovery. The modelled probability of persistence (PR) was 8%, equivalent to roughly three to four patients.

Among the 29 immediate-onset FNP cases, 13 (0.6%) remained unresolved, whereas 3 (0.1%) of the 18 late-onset cases still had deficits at final follow-up. The MPT for immediate-onset FNP exceeded that of late-onset FNP by 107 days (p < 0.05; Fig. 3b), indicating a markedly prolonged recovery course in the early-onset cohort.

The analysis also considered 37 patients who underwent vestibular-schwannoma surgery: 19 (51.4%) developed post-operative FNP, and 10 of these recovered fully. Although the incidence and persistence rates differed from those after other middle-ear procedures, the CPF curves showed no statistically significant difference (p = 0.385).

### Audiological measures

Pure-tone audiograms from 1,597 patients were analysed in order to evaluate bone-conduction (BC) hearing thresholds. Audiometric data were unavailable in cases of pre-operative functional deafness, in children younger than five years of age, or during emergency procedures.

**Fig. 4:**
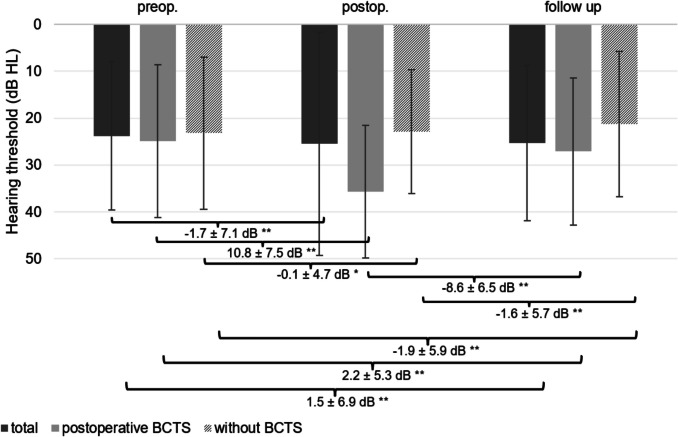
Bone-conduction hearing thresholds (PTA:BC_4_) at three time points (preoperative, postoperative, and follow-up) in the total patient cohort (n = 1,550) as well as in patients with (n = 293) and without (n = 1,257) postoperative bone-conduction threshold shift (BCTS). Patients without BCTS showed a small but significant improvement at follow-up (−1.9 ± 5.9 dB; p < 0.001), while BCTS patients experienced an deterioration (–10.8 ± 7.5 dB) with recovery at follow-up (residual deficit: 2.2 ± 5.3 dB; p < 0.001). *Bars represent mean values* ± standard deviation. * p < 0.05, **p < *0.001*

Figure 4 summarises the pre- and post-operative hearing outcomes, stratified into three groups:

- All patients (n = 1,550)
- Patients with post-operative BCD (n = 293)
- Patients without BCD (n = 1,257)

Among patients without threshold shift in BC, the four-frequency pure-tone average for BC at 0.5, 1, 2 and 4 kHz (PTA-BC_4_) improved by 1.9 ± 5.9 dB at follow-up relative to pre-operative values (p < 0.001).

Among the cohort that exhibited a postoperative decline in bone-conduction (BC) thresholds, the immediate mean loss measured 10.8 ± 7.5 dB. At follow-up (between 6 to a maximum of 24 months postoperatively), thresholds improved by 8.6 ± 6.5 dB (p < 0.001), yielding a residual deficit of 2.2 ± 5.3 dB relative to pre-operative baselines (p < 0.001).

## Discussion

### A shift in outcome assessment: Integrating complication dynamics

Traditionally, surgical outcomes have been evaluated primarily in terms of success, benefit, or improvement. This one-dimensional perspective can contribute to the under-reporting of complications, because the term complication still carries a negative connotation. To enhance the quality of care in otologic surgery, a structured, standardised approach to complications—covering their incidence, severity, and course (resolution versus persistence)—is essential. Recognising this unmet clinical need and defining standardised parameters will foster more accurate reporting and enable earlier intervention in post-operative complications.

### Heterogeneity in reporting complications

The literature on complications after middle-ear surgery is highly heterogeneous, owing chiefly to variations in.

1. surgical techniques studied (e.g. tympanoplasty, stapesplasty, cochlear implantation) [8, 19, 20];
2. types of complications reported (e.g. dizziness, hearing loss, facial-nerve palsy) [2, 8, 20–26]; and
3. follow-up duration and data-collection methods.

Reported complication rates range from 1.4% to 35% [2, 8, 20–26], mirroring differences in definitions, inclusion criteria and methodology. Most studies examine a single technique rather than providing a comprehensive complication analysis, making direct comparisons difficult.

By contrast, the present investigation offers a three-year, surgery-independent evaluation of complications and records an overall rate of 25%. BC threshold shift was the most common event, affecting 14% of patients (293 of 2,120). This broad approach lays the groundwork for future subgroup analyses that can differentiate the risk profiles of specific surgical procedures.

### Dynamic characterization of complications using CPF

The Complication Persistence Function (CPF) provides a statistical framework for describing the resolution and persistence dynamics of complications over time. In contrast to the extant literature, which has thus far focused exclusively on incidence rates, the CPF enables the analysis of.

- *complication-persistence prediction*, even for patients with incomplete follow-up (e.g., those lost to follow-up), and
- *time-dependent recovery patterns*, quantified by metrics such as the median persistence time (MPT) and the plateau onset time (POT).

This statistical approach is particularly valuable in clinical practice, where follow-up duration varies among patients because of relocation, personal decisions, or medical necessity.

### Bone-conduction threshold shift after ear surgery

Post-operative BC threshold shift rates reported in the literature range from 1.2% to 35% [27–29], whereas our study found an intermediate rate of 13.9%. Two main factors explain the variability across studies:

1. Follow-up duration (from a few weeks to several years)
2. Operational definition of BCD (≥ 10 dB vs ≥ 15 dB threshold shifts at selected frequencies)

The findings of this study demonstrate that immediate and late-onset BC threshold shift follow distinct recovery trajectories, which is indicative of different underlying pathophysiological mechanisms:

- Immediate BCD is largely attributable to surgical trauma (e.g., drilling, mechanical manipulation on the ossicular chain).
- Late-onset BCD is more likely to be caused by inflammatory processes (e.g., tissue reaction).

Complete recovery was more frequently observed with late-onset BC threshold shift, supporting the hypothesis that inflammatory deterioration has a better prognosis than surgically induced BC threshold shift.

### Re-evaluating the use of the ABG as an outcome parameter

The air–bone gap (ABG) remains the principal outcome measure in the audiological literature [30, 31]. However, a change in BC thresholds alone can artificially narrow the ABG and thereby misrepresent functional outcomes [9]. Although the 1995 AAO-HNS guidelines already recommended incorporating BC changes into surgical outcome assessments, standardised international reference values are still lacking [6].

In our cohort, bone-conduction threshold shift persisted in 33.3% of cases at the last follow-up, whereas statistical modelling yielded a persistence rate of only 6%. This pronounced discrepancy underscores the importance of analysing complications dynamically over time:

- Incidence rates by themselves may overestimate long-term impact.
- Persistence rates by themselves may underestimate clinical relevance.

Reporting the observed and modelled persistence rates in parallel therefore provides the most accurate assessment of postoperative complications.

### Unexpected post-operative BC improvement

Interestingly, patients without complications, bone-conduction thresholds improved significantly postoperatively. The underlying cause remains unclear, but several mechanisms may contribute:

1. Repeated audiometric testing, which can produce a training effect in psychophysical measurements.
2. Transient conductive alterations—for example, ear-canal packing material or soft-tissue swelling—that modify the acoustic environment.
3. Other mechanisms analogous to the threshold shifts seen after stapesplasty, although this explanation seems unlikely in our cohort [32].

Further studies are warranted to elucidate this phenomenon.

### Facial Nerve Palsy (FNP) after ear surgery

Post-operative FNP rates reported in the literature range from 0.2% to 5% [8, 25, 33, 34]; in our series the incidence was 2.2% (47 of 2,120 cases). As with BC threshold shift, immediate and late-onset FNP followed distinct recovery trajectories:

- Immediate FNP recovered more slowly and carried a higher probability of persistence.
- Late-onset FNP resolved almost completely, suggesting an inflammatory rather than traumatic aetiology.

Although FNP occurred in 51.4% of vestibular-schwannoma procedures, the associated CPF curves did not differ significantly from those of middle-ear surgeries (p = 0.385). Given the unique prognosis of intra-operative facial-nerve injury during schwannoma resection, dedicated subgroup analyses are warranted. Future studies should aim to identify risk factors and reliable predictors of FNP outcome.

## Limitations

This study constitutes an initial step toward standardising parameters for complication assessment. Several limitations, however, must be acknowledged:

- Single-centre design, which limits the generalisability of the findings.
- Absence of universally accepted classification systems for ear-specific complications; the distinction between an expected post-operative course and a true complication therefore relied on clinical judgement.
- Planned subgroup analyses are still pending; these will be required to refine the proposed parameters and to evaluate their clinical utility.

## Conclusion

Because complication reporting is still heterogeneous, we present the first data from a prospective, longitudinal complication registry established at a tertiary otorhinolaryngology–head and neck surgery referral centre. In the absence of standardised guidelines, our findings.

- define core documentation items that can be embedded in everyday clinical workflows, and
- illustrate the Kaplan–Meier estimator as a pragmatic tool for time-resolved complication analysis.

We propose replacing the traditional dichotomous view of complications (present vs absent) with a continuous, time-dependent description based on the Complication Persistence Function (CPF). The CPF provides a generic framework for any adverse event and yields clinically intuitive parameters such as the median persistence time (MPT) and the probability of residual long-term morbidity (PR). Broad adoption of this methodology would constitute a major step toward standardised complication reporting, strengthen internal quality assurance, and improve inter-centre comparability and knowledge exchange at both national and international levels.
